# Expression Profiles, Characterization and Function of *HbTCTP* in Rubber Tree (*Hevea brasiliensis*)

**DOI:** 10.3389/fpls.2016.00789

**Published:** 2016-06-07

**Authors:** Zhi Deng, Jiangshu Chen, Julie Leclercq, Zhuangzhi Zhou, Changren Liu, Hui Liu, Hong Yang, Pascal Montoro, Zhihui Xia, Dejun Li

**Affiliations:** ^1^Key Laboratory of Biology and Genetic Resources of Rubber Tree, Ministry of Agriculture, Rubber Research Institute, Chinese Academy of Tropical Agricultural SciencesDanzhou, China; ^2^Hainan Key Laboratory for Sustainable Utilization of Tropical Bioresources, College of Agriculture, Hainan UniversityHaikou, China; ^3^CIRAD, UMR AGAPMontpellier, France; ^4^State Key Laboratory of Plant Genomics and National Center for Plant Gene Research, Institute of Genetics and Developmental Biology, Chinese Academy of SciencesBeijing, China

**Keywords:** expression analyses, *Hevea brasiliensis*, stress/hormone response, DNA protection activity, TCTP, TPD

## Abstract

As a highly conserved protein, the translationally controlled tumor protein (TCTP) carries out vital roles in various life processes. In rubber tree, two TCTP genes, *HbTCTP* and *HbTCTP1*, were cloned, but only *HbTCTP1* was studied in details. In this study, *cis*-acting regulatory elements, expression patterns, subcellular localization, interacting proteins, and antioxidant activity of *HbTCTP* were systematically analyzed. Besides the common *cis*-acting regulatory elements, *HbTCTP* promoter also harbored various known *cis*-elements that respond to hormone/stresses. Being consistent with the aforementioned results, *HbTCTP* was regulated by drought, low temperature, high salt, ethylene (ET), wounding, H_2_O_2_, and methyl jasmonate (MeJA) treatments. *HbTCTP* was expressed throughout different tissues and developmental stages of leaves. In addition, *HbTCTP* was associated with tapping panel dryness (TPD). HbTCTP was localized in the membrane, cytoplasm and the nucleus, and interacted with four proteins rubber elongation factor (REF), 17.5 kDa heat shock family protein, annexin, and REF-like stress related protein 1. Being similar to HbTCTP1, HbTCTP also indicated antioxidant activity in metal-catalyzed oxidation (MCO) system. Our results are useful for further understanding the molecular characterization and expression profiles of *HbTCTP*, but also lay a solid foundation for elucidating the function of *HbTCTP* in rubber tree.

## Introduction

The translationally controlled tumor protein (TCTP) is a highly conserved protein, and it is widely expressed in all eukaryotic organisms (Bommer and Thiele, 2004). Since Yenofsky et al. (1983) reported the first TCTP in mouse, the TCTPs have been cloned and characterized in different organisms. In animals, the TCTPs were reported to be associated with several biological processes such as cell growth, cell cycle progression, differentiation, malignant transformation, protection against various stress conditions and apoptosis (Amson et al., 2013).

In contrast to animals, the studies on plant TCTPs are very limited. The first plant *TCTP* gene was cloned in *Medicago sativa* (Pay et al., 1992). Currently, *TCTP* genes have been isolated from different plants species. Besides being related to plant growth and development (Lu et al., 2007; Brioudes et al., 2010; Nakkaew et al., 2010; Qin et al., 2011), *TCTPs* were also involved in a wide range of stimulus response, such as darkness (Sage-Ono et al., 1998), aluminum (Ermolayev et al., 2003), pathogens (Jones et al., 2006; Li et al., 2010b), NaCl (Vincent et al., 2007; Cao et al., 2010), mercury (Wang et al., 2012), heat, cold, and drought (Kim et al., 2012; Li et al., 2013), as well as growth regulators such as auxins, ABA (Berkowitz et al., 2008; Cao et al., 2010; Kim et al., 2012), and methyl jasmonate (MeJA) (Li et al., 2013). Interestingly, the TCTPs have been detected in castor and winter squash phloem saps, suggesting that the TCTPs might be involved in regulating the destination-selective long-distance movement of phloem proteins by interacting with phloem proteins (Barnes et al., 2004; Aoki et al., 2005; Hinojosa-Moya et al., 2006). Recently, lupin and pumpkin *TCTP* mRNA have also been found in the phloem sap transcriptome and phloem sap exudates, respectively (Rodriguez-Medina et al., 2011; Hinojosa-Moya et al., 2013).

TCTP varies in its gene number among different organisms. Mammals have more than two TCTP-like sequences, while plants and fungi usually contain one or two *TCTP* genes (Hinojosa-Moya et al., 2008). Based on this wide range of functions and the different TCTP versions, Gutiérrez-Galeano et al. (2014) suggested that TCTPs might take place function specialization in plants with two members. They further proposed that there were two groups of *TCTP* genes, *AtTCTP1*-like and *CmTCTP*-like clades, and their respective functions might be inferred from their corresponding groups.

There are two *TCTP* genes identified in rubber tree (Li et al., 2013). Liang et al. (2009) firstly reported that *HbTCTP* (GenBank accession: FJ156098) was constitutively expressed in latex, leaves and barks and induced by ethylene (ET) treatment. Deng et al. (2012) further analyzed the gene structure and developed molecular markers of *HbTCTP*. Li et al. (2013) cloned another *TCTP* gene (*HbTCTP1*) in rubber tree. *HbTCTP1* was expressed throughout different tissues and developmental stages of leaves. Besides being related to tapping panel dryness (TPD), *HbTCTP1* was regulated by drought, low temperature, high salt, ET, wounding, H_2_O_2_, and MeJA. HbTCTP1 possessed supercoiled DNA protection activity in metal-catalyzed oxidation (MCO) system. The aforementioned results suggested that *HbTCTP1* was a multifunctional gene and associated with hormone/stress response and TPD. However, the other *TCTP* gene, *HbTCTP*, was not studied and reported in details until now. Here, we further analyzed expression patterns, *cis*-regulatory elements of *HbTCTP*, as well as interacting proteins, subcellular location, and antioxidant activity of HbTCTP protein. Our results not only are useful for further understanding the molecular characterization and expression profiles of *HbTCTP*, but also lay a solid foundation for elucidating *HbTCTP* function in rubber tree.

## Materials and methods

### Plant materials

Reyan 8-79, a high-yielding rubber tree clone, was planted at the experimental farm of Chinese Academy of Tropical Agricultural Sciences. The plant tissues, latex, male and female flowers, anthers, leaves and barks were separately collected from three 17-year-old rubber trees regularly tapped on the s/2 d/3 system with 1% ET stimulation for the past 11 years, and each rubber tree was referred as one biological replicate. According to Hao and Wu (2000), 6-year-old virgin trees were applied with ET, H_2_O_2_ and MeJA treatments prior to the first tapping. The wounding treatment was performed according to the method provide by Tang et al. (2010). One-year old seedlings of Reyan 7-33-97 were separately treated with drought (16% PEG), low temperature 8(8°C), and high salt (1M NaCl), and the leaves from five treated and five control (1-year old seedlings of Reyan 7-33-97 were grown at normal condition without any treatments including drought, low temperature, or high salt) materials were separately collected and equivalently pooled at different times for total RNA extraction. The leaves from each five seedlings were referred as one biological replicate. The leaves of different developmental stages were separately harvested from three 6-year-old Reyan 8-79, and each rubber tree was referred as one biological replicate. The trees with a partial or complete stoppage of latex flow are defined as the initial and advanced stages of TPD trees, respectively. In this study, the latex and barks were collected from the TPD trees at initial stage. The latex and barks from five healthy and five TPD rubber trees were separately collected and equivalently pooled for total RNA exaction, and the samples from each five trees were referred as one biological replicate. All samples for real-time RT-PCR experiments were harvested with three biological replicates.

### RNA and DNA extraction

Total RNAs from different tissues were prepared according to Xu's method (Xu et al., 2010), and then treated with RNase-free RQ1 DNase (Promega, USA). The genomic DNA was isolated from the young leaves with the CTAB method (Porebski et al., 1997). The quantity and quality of RNA and DNA were determined by agarose gel electrophoresis and the spectrophotometer.

### Cloning and analyses of *HbTCTP* promoter

To obtain the promoter sequence of *HbTCTP*, the PCR primers were designed according to the sequences of contig338249 (GenBank accession: AJJZ010271614.1) (Rahman et al., 2013). The promoter sequence of *HbTCTP* was isolated by PCR amplification using GenomeWalker universal kit (Clontech, USA). The primary PCR was performed with the outer adaptor primer (AP1) and the outer gene-specific primer 5′-CAGAATCACTCACCCAAACG-3′ using Advantage2 polymerase mix (Clontech, USA). The secondary PCR was performed with the nested adaptor primer (AP2) and the nested gene-specific primer 5′-CAGATCCCCATGTACACAAC-3′. The PCR product was cloned into the pMD18-T vector and sequenced. The *cis*-acting regulatory elements within the promoter of *HbTCTP* were predicted using PLACE (https://sogo.dna.affrc.go.jp/cgi-bin/sogo.cgi?lang=en&pj=640&action=page&page=newplace).

### Real-time RT-PCR

First-strand cDNA was obtained using ReverAid™ first strand cDNA synthesis kit (Thermo Scientific, USA). In each real-time RT-PCR reaction, the gene-specific primers were used, and *18S rRNA* gene (GenBank accession: AB268099) was used as the internal reference according to Tang et al. (2010). The forward (F) and reverse (R) primers are the following: for *18S rRNA* (F 5′-GCTCGAAGACGATCAGATACC-3′ and R 5′-TTCAGCCTTGCGACCATAC-3′); for *HbTCTP* (F 5′-TTGTGGATCGATCCGAGAGA-3′ and R 5′-CAACCC ACTTCCCCTCAACT-3′). Real-time RT-PCR was performed with the LightCycler 2.0 system (Roche Diagnostics, Germany) and SYBR premix Ex Taq™ II (Takara, Japan). The reactions were carried out as follows: 30 s at 94°C for denaturation, followed by 45 cycles of 94°C for 5 s, 60°C for 20 s, and 72°C for 20 s. The relative abundance was calculated by the LightCycler Relative Quantification Software 4.05. The specificity of each primer pairs was verified by the melting curves and sequencing the PCR products. All real-time RT-PCR experiments described here were reproduced with three biological replicates, and the values were presented as mean ± S.D. The statistical analysis was performed using SPSS software version 19.0 (IBM, Armonk, New York) and one-way ANOVA with the Student-Newman-Keuls test was used for comparison of *HbTCTP* expressions across different tissues and development stage. The *t*-test was performed for comparison of *HbTCTP* expressions under the treatments of stresses and hormones, or healthy and TPD rubber trees.

### Subcellular localization of HbTCTP

To determine its subcellular localization, the ORF of *HbTCTP* was cloned into the vector CD3-1103 (TAIR Accession: 5016231514). In this construct, the gene was in-frame fused at its 5′ end to an enhanced Yellow Fluorescent Protien (eYFP) gene. The forward and reverse primers were 5′-CTCAAGCTTCGAATTCTATGTTGGTCTATCAGGATTTGCT-3′ and 5′-CTAGATCAGGTGGATCCGCATTTGACCTCCT TCAAAGCA-3′, respectively. The PCR-amplified fragment was ligated into the vector basing on the method of homologous recombination. The resultant construct was transformed into rice protoplasts for transient expression, following the methods described by Bart et al. (2006). The fluorescent signals were examined with a laser confocal microscope (Leica TCS SP5), stimulated by light of 512 nm wavelength [To indicate cell nucleus, the nuclear mCherry expression vector CD3-1106 (TAIR Accession: 5016231517) was optionally cotransformed into rice protoplasts together with the construct. mCherry fluorescence was stimulated by light of 585 nm wavelength].

### Yeast two-hybrid screen and assays

HybriZAP®-2.1 two-hybrid predigested vector/gigapack® cloning kit (Stratagene, CA) was used to screen the proteins interacting with HbTCTP in the study. The coding sequence of *HbTCTP* was cloned into the bait vector pBD-GAL4 Cam. *Saccharomyces cerevisiae* strain YRG-2 were first transformed with the bait pBD-HbTCTP and subsequently transformed with latex cDNA library plasmid DNA by the lithium acetate method. The transformants were plated onto SD medium lacking leucine, tryptophan and histidine (SD/-Leu/-Trp/-His). The colonies growing on selection media were restreaked onto SD/-Leu/-Trp/-His, transferred to the filter paper, and assayed for β-galactosidase (LacZ) activity by the filter lift assay. The LacZ-positive colonies growing on SD/-Leu/-Trp/-His medium were considered putative positive clones. The plasmids were isolated from these yeast colonies, transformed, and amplified in the *E. coli* XL1-Blue strain. The isolated plasmids were then transformed back into YRG-2 cells either alone or in combination with control plasmids. The retransformation assays were able to elicit expression of the two reporter genes only in the presence of pBD-HbTCTP bait, but not alone or in combination with control plasmids, were considered true positives. The validated positive clones were selected to be sequenced.

### Purification and MCO assay of HbTCTP fusion protein

The coding sequences of *HbTCTP* with the introduced *Eco*R I and *Bam*H I sites were ligated into pET28a plasmid. The correct pET28a-HbTCTP was confirmed by sequencing and transferred into *E. coli* strain BL21 (DE3). In LB medium with 100 mg/L kanamycin, the *E. coli* BL21 cells containing pET28a-HbTCTP or pET28a plasmids were separately grown at 37°C overnight. One mL transformants cultured overnight were inoculated into 200 mL fresh LB medium with 100 mg/L kanamycin. After the OD600 value of the transformants reached 0.4, the transformants were further incubated at 37°C for ~3 h with 1 mM isopropyl-β-D-thiogalactopyranoside (IPTG) induction. To obtain HbTCTP protein, the pET28a-HbTCTP was purified and substituted by Zeba™ Desalt Spin Columns (Thermo Scientific Pierce, USA). The MCO assay was employed to determine whether HbTCTP could protect supercoiled DNA according to the method described by Li et al. (2004). Fifty microliters reaction mixtures contain 33 μM FeCl_3_, 3.3 mM DTT and 200 μg/mL purified HbTCTP or HbTCTP1 proteins. The pUC19 supercoiled DNA (300 ng) were added to each reaction and incubated further at 37°C for 2 h. The DNA protection was evaluated with 10 μL reaction samples by 1% agarose gel electrophoresis. In addition, bovine serum albumin (BSA) was also assayed in similar conditions as controls.

### Degradome data

According to German et al. (2009), a library with all degraded mRNA was constructed in rubber tree. After sequencing, analysis with the Cleaveland pipeline, slicing sites of mRNA by small RNA are detected. Degradome data were provided by the DEBA *Hevea* project funded by Institut Français du caoutchouc. Degradome data were obtained according to an adapted protocol from German et al. (2009) and analyzed by the CLEAVELAND pipeline developed by Addo-Quaye et al. (2009).

## Results

### Hormone/stresses respond-related *cis*-elements identified in *HbTCTP* promoter

Using the PCR-based Genome Walking method, we obtained a 1201-bp promoter sequence of *HbTCTP* (GenBank accession: KX179468). The *cis*-acting regulatory elements within *HbTCTP* promoter were predicted by PLACE software. Besides the common *cis*-acting regulatory elements including TATA boxes and CAAT boxes, the *HbTCTP* promoter also harbored various known *cis*-elements associated with hormone response [ethylene, abscisic acid (ABA), gibberellin (GA), salicylic acid (SA), and auxin responses], stress response (anaerobically induced, disease resistance, copper, CO_2_, pathogenesis and salt-induced, light, wounding, UV-B irradiation, heat shock, water, and pro- or hypo-osmolarity responses), as well as stress and hormone responses (drought and ABA, dehydration, ABA and cold, GA and sugar). In addition, some special *cis*-acting regulatory elements were also found in *HbTCTP* promoter, such as phytochrome regulated element, and sugar response element, etc…(Supplementary Table S1). The results indicated that *HbTCTP* expression might be involved in stress/hormone response.

### Expression analysis of *HbTCTP*

**Figure 1:**
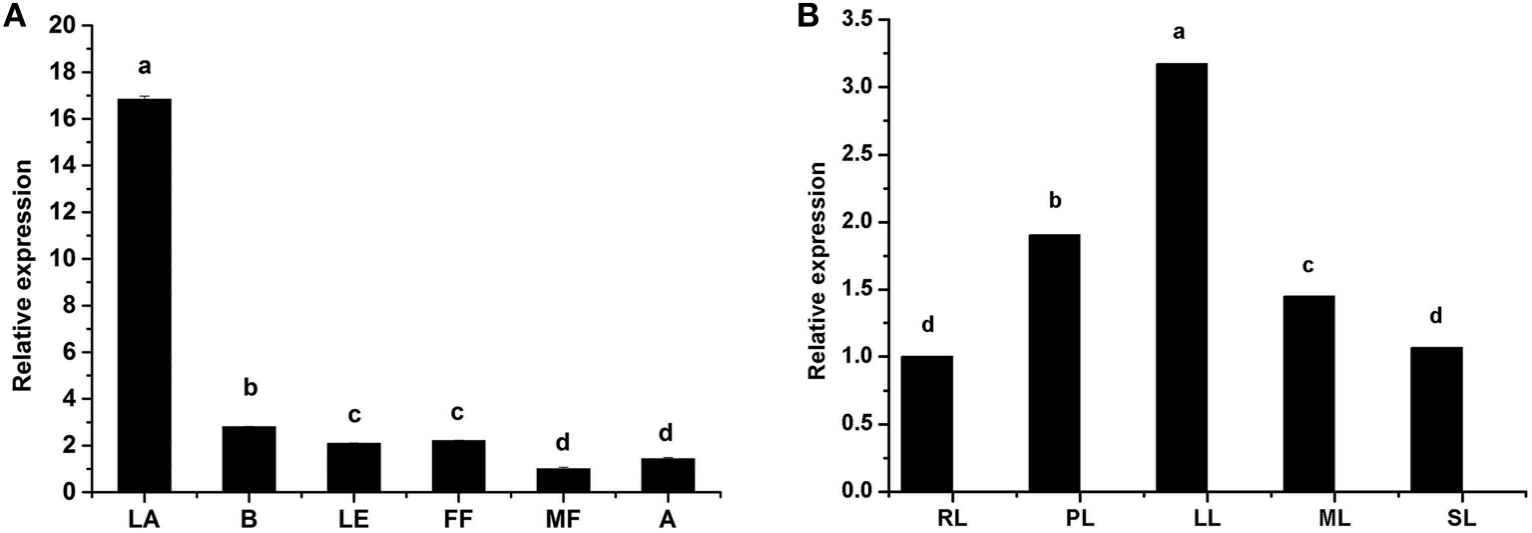
**Real time RT-PCR analyses of *HbTCTP* expression patterns in different tissues and developmental stages of leaves**. Panels **(A,B)** represent *HbTCTP* expression profiles in different tissues and developmental stages of leaves, respectively; B, LA, LE, FF, MF, and A represent barks, latex, leaves, female flowers, male flowers, and anthers, respectively. RL, PL, LL, ML, and SL separately are red leaves, pale-green young, light young, mature, and senescence leaves. *18S rRNA* is used as the internal control for real time RT-PCR analyses. The standard bars were obtained from at least three independent replications (different RNA preparations from different biological replicates) and the results were given as mean ± S.D. The different letters indicate significant difference at *P* < 0.05.

We systematically analyzed *HbTCTP* expression by real time RT-PCR. As shown in Figure 1A, *HbTCTP* was highly expressed in latex, followed by barks, female flowers, leaves, anthers and male flowers. Gébelin et al. (2012) predicted that eleven contigs encoding rubber tree *TCTP* genes could be targeted by miRNA. Therefore, we set out to detect whether this occurs to *HbTCTP*. As shown in Table 1, three contigs (CL1994Contig3, CL18Contig4, and CL18Contig13) being identified to *HbTCTP* are experimentally validated by degradome analysis in at least two tissues. In addition, *HbTCTP* expression indicated a significant change in different leaf development stages, with the highest level in light young leaves, the lowest in red leaves (Figure 1B).

**Table 1:** **The information of *HbTCTP* experimentally validated by degradome analysis**.

***HbTCTP***							
SiteID	CL1994Contig3:25	CL1994Contig3:25	CL18Contig4:316	CL18Contig4:316	CL18Contig13:280	CL18Contig13:280	CL18Contig13:280
Query	acc_635132_22	acc_635132_22	acc_298609_20	acc_81739_20	acc_298609_20	acc_298609_20	acc_298609_20
Query GenBank accession	KX230767	KX230767	KX230768	KX230769	KX230768	KX230768	KX230768
miRNA homology	ppt-miR1023b-3p	ppt-miR1023b-3p	ptc-miRf12236-akr	ptc-miRf12236-akr	ptc-miRf12236-akr	ptc-miRf12236-akr	ptc-miRf12236-akr
Transcript	CL1994Contig3	CL1994Contig3	CL18Contig4	CL18Contig4	CL18Contig13	CL18Contig13	CL18Contig13
Transcript GenBank accession	KX230764	KX230764	KX230765	KX230765	KX230766	KX230766	KX230766
TStart	12.0	12.0	303.0	303.0	267.0	267.0	267.0
TStop	34.0	34.0	325.0	325.0	289.0	289.0	289.0
TSlice	25.0	25.0	316.0	316.0	280.0	280.0	280.0
MFEperfect	−34.6	−34.6	−31.5	−31.5	−31.5	−31.5	−31.5
MFEsite	−22.7	−22.7	−20.6	−20.6	−20.6	−20.6	−20.6
MFEratio	0.65606936416185	0.65606936416185	0.65396825396825	0.65396825396825	0.65396825396825	0.65396825396825	0.65396825396825
AllenScore	10.0	10.0	11.5	11.5	11.5	11.5	11.5
Paired	3-5,32-30;8-18,27-17;19-22,15-12	3-5,32-30;8-18,27-17;19-22,15-12	4-17,322-309;18-20,305-303	4-17,322-309;18-20,305-303	4-17,286-273;18-20,269-267	4-17,286-273;18-20,269-267	4-17,286-273;18-20,269-267
Unpaired	1-2,34-33[UP5];6-7,29-28[SIL];x-x,16-16[BULt]	1-2,34-33[UP5];6-7,29-28[SIL];x-x,16-16[BULt]	1-3,325-323[UP5];x-x,308-306[BULt]	1-3,325-323[UP5];x-x,308-306[BULt]	1-3,289-287[UP5];x-x,272-270[BULt]	1-3,289-287[UP5];x-x,272-270[BULt]	1-3,289-287[UP5];x-x,272-270[BULt]
Structure	((((.(((((((((((.(((.&.))).)))))))))))-))))	((((.(((((((((((.(((.&.))).)))))))))))-))))	(((…((((((((((((((…&…))))))))))))))—)))	(((…((((((((((((((…&…))))))))))))))—)))	(((…((((((((((((((…&…))))))))))))))—)))	(((…((((((((((((((…&…))))))))))))))—)))	(((…((((((((((((((…&…))))))))))))))—)))
Sequence	CUCUCGCGCUCUCUUUUU UUCCC& AAGAAUUGAAGAGAGUGU-AGAG	CUCUCGCGCUCUCUUUUUUUCCC&AAGAAUUGAAGAGAGUGU-AGAG	CCAAAUUUUUGGGGGGGGGAGAG&CAUUCCUCUUCCUAAAA—UGG	CCAAAUUUUUGGGGGGGGGAGAG&CAUUCCUCUUCCUAAAA—UGG	CCAAAUUUUUGGGGGGGGGAGAG&CAUUCCUCUUCCUAAAA—UGG	CCAAAUUUUUGGGGGGGGGAGAG&CAUUCCUCUUCCUAAAA—UGG	CCAAAUUUUUGGGGGGGGGAGAG&CAUUCCUCUUCCUAAAA—UGG
DegradomeCategory	2	3	4	4	4	4	4
DegradomePval	0.057064826793158	0.043840735949404	0.043696995831537	0.040822193474199	0.079977935468352	0.085484564218372	0.11412318662399
Librairy	bark	leaf	root	latex	latex	root	leaf
Length	22	22	20	20	20	20	20

SiteID, a unique name for a putative splicing site in the form of “[transcript]:[slice_site]”; Query, small RNA accession; miRNA homology, column added by the authors with the best hit after blasting the small RNA sequence against PMRD database (Zhang et al., 2010); Tstart, one-based start position of the alignment within the transcript; Tstop, one-based stop position of the alignment within the transcript; Tslice, one-based position of the alignment opposite position 10 of the query; MFEperfect, minimum free energy of a perfectly matched site (approximate); MFEsite, minimum free energy of the aligment in question; MFEratio, MFEsite/MFEperfect; AllenScore, penalty score calculated by Allen (Allen et al., 2005); Paired, string representing paired position in the query and transcript; Unpaired, string representing unpaired positions in the query and transcript; Structure, aligned secondary structure; Sequence, aligned sequences of transcript and query; Degradome category, category 4: just one read at this position; category 3: >1 read, but below or equal to the average depth of coverage on the transcript; category 2: >1 read, equal to the average depth of coverage on the transcript; category 1: >1 read, equal to the maximum of the average depth of coverage on the transcript when there is >1 position at maximum value; category 0: >1 read, equal to the maximum of the average depth of coverage on the transcript when there is just one position at maximum value; DegradomePval, p-value for this degradome hit; Library, column added by the authors, name of the tissue from which degradome library was sequenced; Length, column added by the authors, small RNA length in nucleotide.

**Figure 2:**
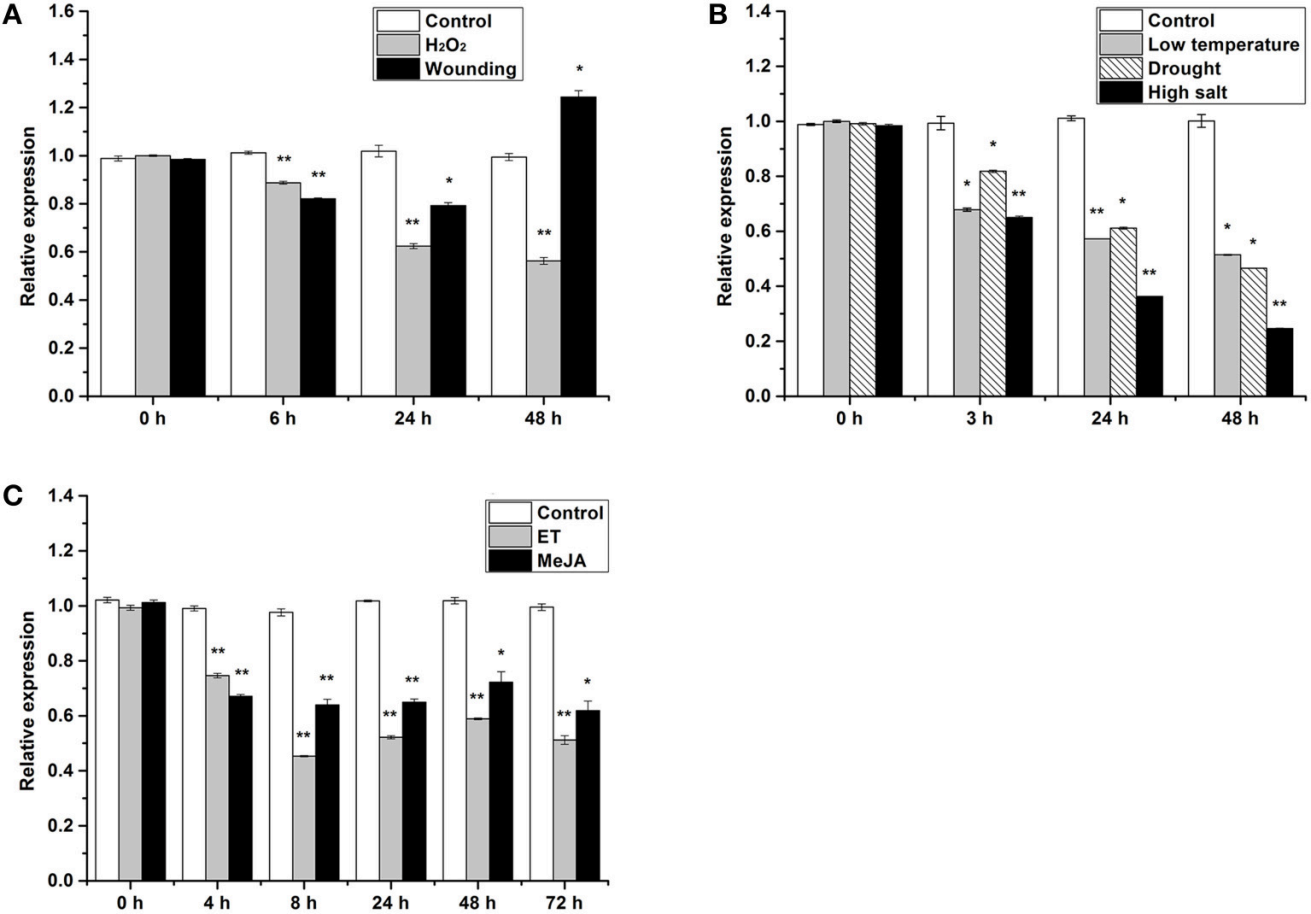
**Real time RT-PCR analyses of *HbTCTP* expression patterns under different treatments**. Panels **(A–C)** represent real time RT-PCR analyses of *HbTCTP* expression profiles under H_2_O_2_ and wounding; low temperature, drought, and high salt; ET and MeJA treatments, respectively; *18S rRNA* is used as the internal control for real time RT-PCR analyses. The standard bars were obtained from at least three independent replications (different RNA preparations from different biological replicates) and the results were given as mean ± S.D. Compared with the control, one asterisk shows significant difference with a *P* < 0.05, and two asterisks show very significant difference with a *P* < 0.01.

*HbTCTP* expression was also analyzed under several treatments including drought, low temperature, high salt, ET, wounding, H_2_O_2_ and MeJA. As shown in Figure 2, *HbTCTP* was regulated by all the treatments, but its expression profiles widely varied depending on the different treatments. The statistical analyses showed that the changes of *HbTCTP* expression indicated significant differences under all treatments. Under drought, low temperature, high salt, and H_2_O_2_ treatments, *HbTCTP* transcripts steadily decreased (Figures 2A,B). Under ET and MeJA treatment, the *HbTCTP* was totally downregulated, but indicated slight oscillations in different times (Figure 2C). Under wounding treatment, *HbTCTP* transcripts were decreased at 6 h after treatment, and then upregulated at 48 h (Figure 2A). In contrast, *HbTCTP* did not significantly change in their corresponding controls (Figure 2). These results suggested that *HbTCTP* might be associated with ET, MeJA and stress responses in rubber tree.

**Figure 3:**
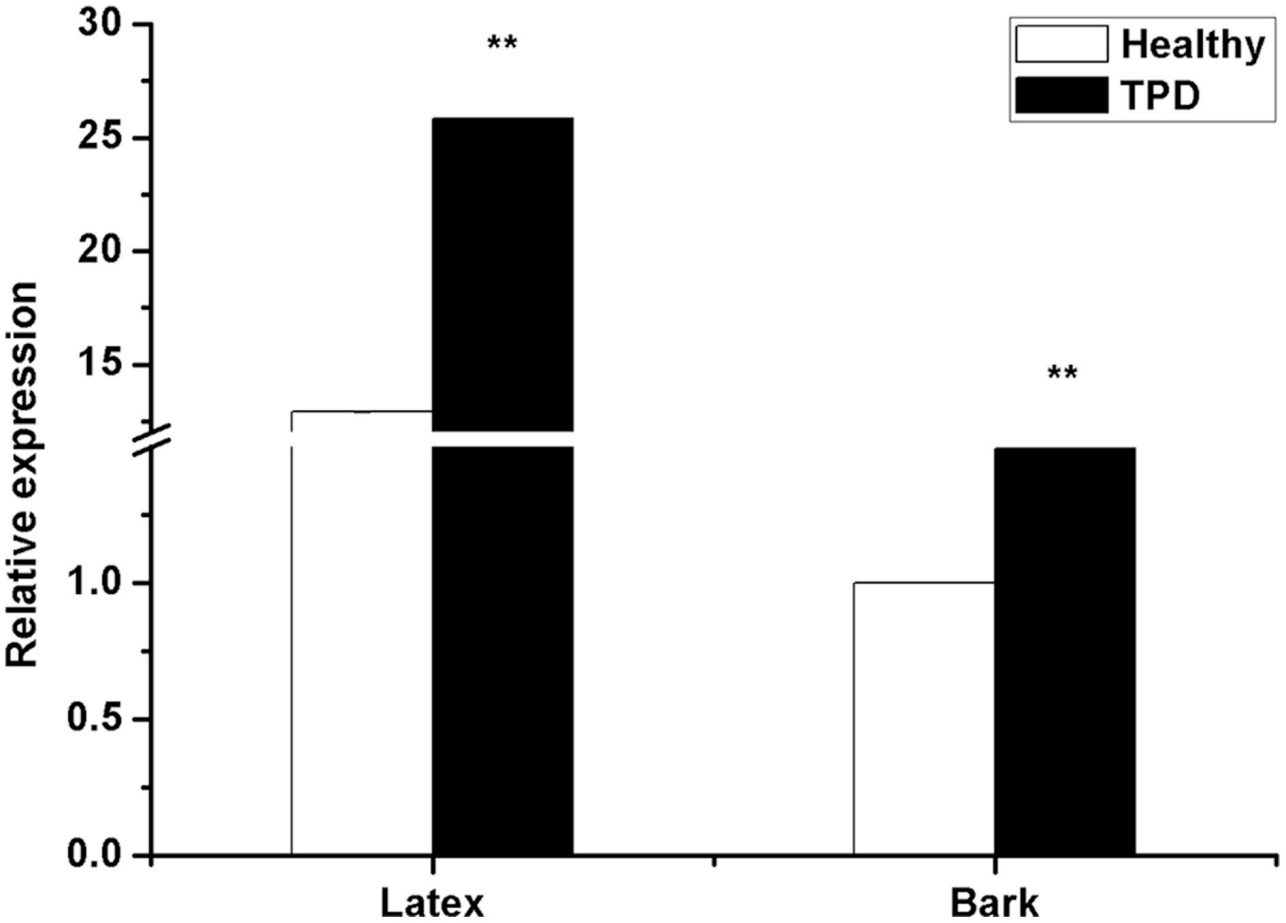
**Real time RT-PCR analyses of *HbTCTP* expression in healthy and TPD rubber trees**. *18S rRNA* is used as the internal control for real time RT-PCR analyses. The standard bars were obtained from at least three independent replications (different RNA preparations from different biological replicates) and the results were given as mean ± S.D. Compared with the healthy rubber tree, two asterisks show very significant difference with a *P* < 0.01.

Li et al. (2013) reported that *HbTCTP1* transcripts were upregulated in healthy rubber tree, suggesting that *HbTCTP1* was associated with TPD; therefore, *HbTCTP* expression was also investigated in healthy and TPD trees. As shown in Figure 3, the *HbTCTP* transcripts were higher in TPD rubber tree than healthy one. The statistical analyses indicated that the changes of *HbTCTP* expression had significant differences between healthy and TPD rubber trees. The results suggested that *HbTCTP* might be associated with TPD processes in rubber tree.

### HbTCTP located in whole cell

**Figure 4:**
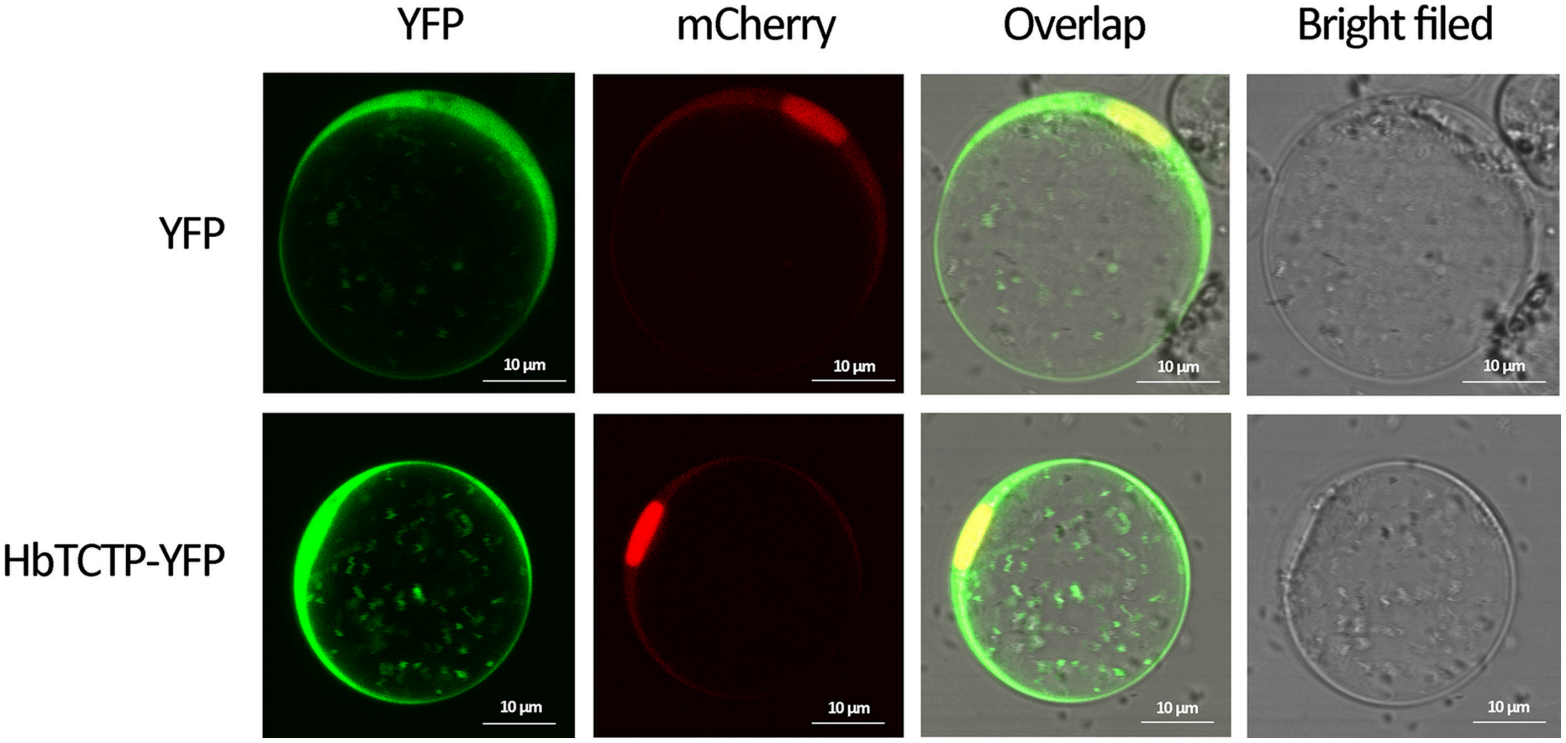
**Subcellular localization of HbTCTP-YFP fusion protein in rice protoplasts**.

To investigate the subcellular localization of HbTCTP protein, we transiently expressed HbTCTP: eYFP fusion protein in rice protoplasts and examined with a laser confocal microscope. As shown in Figure 4, the fluorescent signals of the fusion protein were visualized in the membrane, the cytosol and the nucleus, as similar as those of eYFP alone. The results suggested that HbTCTP has no preference in subcellular localization.

### Identification of HbTCTP-interacting proteins

**Figure 5:**
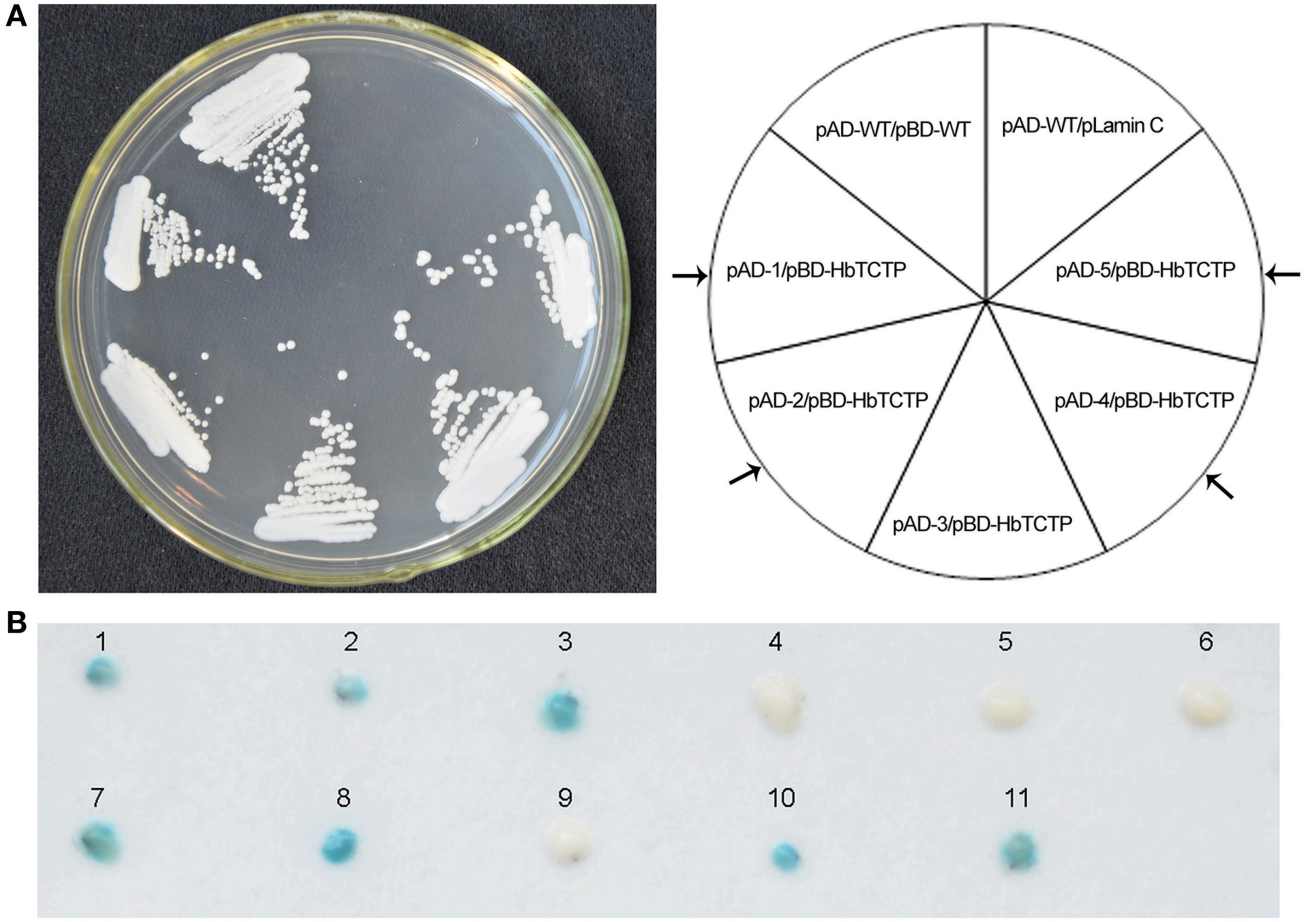
**Identification of the interaction protein of HbTCTP by yeast two-hybird system. (A)** Yeast cells transformed with bait and prey vectors were streaked onto SD/-Leu/-Trp/-His. pAD-WT/pBD-WT and pAD-WT/pLamin C were used as positive and negative controls, respectively. **(B)** β-Galactosidase assays. The numbers 1–11 indicate yeast cells transformed with the following plasmid combinations: 1, pAD-WT/pBD-WT; 2, pAD-MUT/pBD-MUT; 3, pGAL4; 4, pAD-WT/pLamin C; 5, pAD-MUT/pLamin C; 6, pLamin C; 7, pAD-1/pBD-HbTCTP; 8, pAD-2/pBD-HbTCTP; 9, pAD-3/pBD-HbTCTP; 10, pAD-4/pBD-HbTCTP; 11, pAD-5/pBD-HbTCTP. Arrow indicated that the positive clones.

Next, we screened and identified the HbTCTP-interacting proteins by yeast two-hybrid (Y2H) assay. The initial screening identified five colonies, which were subsequently validated for activating the *LacZ* gene. As shown in Figure 5, only four colonies can activate *LacZ* gene. The plasmid DNAs were isolated from the four colonies and retransformed into the YRG-2 strain to confirm whether the activation is indeed due to the presence of the fusion protein. We observed that all the four plasmids were able to activate the *LacZ* gene only in the presence of pBD-HbTCTP. All the four sequences contained complete open reading frames, and the blastx results indicated that the four sequences (GenBank accession: KX179469–KX179472) encoded different proteins including rubber elongation factor protein, 17.5 kDa heat shock family protein, annexin, and REF-like stress related protein 1.

### HbTCTP indicating *in vitro* supercoiled DNA protection activity

**Figure 6:**
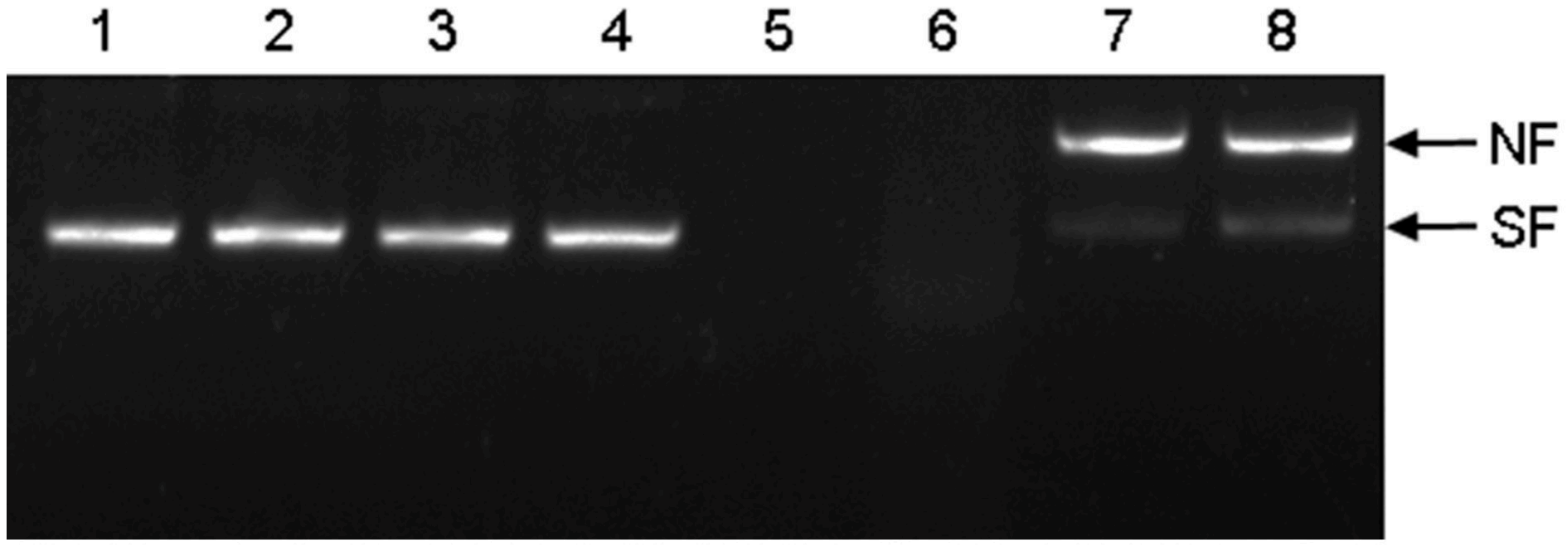
**Protection of supercoiled DNA cleavage by recombinant HbTCTP in a MCO system**. (1) pUC19 without incubation; (2) pUC19 in water and incubated 37°C for 2 h; (3) pUC19 only with FeCl_3_; (4) pUC19 only with DTT; (5) pUC19 with MCO system; (6) pUC19 with BSA (0.6 mg/mL) with MCO system as a negative control; (7) pUC19 with HbTCTP protein (200 μg/mL) with MCO system; (8) pUC19 with HbTCTP1 protein (200 μg/mL) with MCO system. NF, nicked form; SF, supercoiled form of the plasmid.

To analyze *in vitro* supercoiled DNA protection activities, HbTCTP was expressed in *E. coli* BL21 cells and purified. The obtained fusion protein showed an approximately 25.0-kDa band on SDS-PAGE, in agreement with the predicted molecular mass (19.1 kDa for HbTCTP and 6.2 kDa for the His and T7 tags), and was therefore used for DNA protection activity assay. As shown in Figure 6, the MCO components without incubation or separately incubated MCO components did not bring about any damage to supercoiled DNA, whereas the complete nicking of supercoiled DNA occurred in MCO system without HbTCTP protein and with BSA (a negative control). Being consistent with HbTCTP1 identified with supercoiled DNA protection activity (Li et al., 2013), the HbTCTP proteins indicated the protection of supercoiled DNA, suggesting that HbTCTP protein possessed supercoiled DNA protection activity against MCO.

## Discussion

TCTP is a highly conserved protein throughout all the eukaryotes, and it is associated with a wide range of cellular functions including cell growth, cell cycle progression, and protection against various stresses, etc. It is reported that there are variable numbers of *TCTP* genes in different organisms, and plants in general contain one or two *TCTP* genes (Hinojosa-Moya et al., 2008). Rubber tree contains two *TCTP* genes, *HbTCTP* and *HbTCTP1*, but only *HbTCTP1* was analyzed and reported in detail. In this study, *HbTCTP* was systematically studied. Being different from *HbTCTP1, HbTCTP* was highly expressed in latex, which differ from the previous results that there was no difference in the expression of *HbTCTP* among barks, leaves and latex (Liang et al., 2009). With leaf growth and development, *HbTCTP* expression was initially increased, and then decreased. The highest and lowest expression of *HbTCTP* was separately in light young and red leaves, whereas the correspondent in mature and light young leaves as far as *HbTCTP1* is concerned.

Besides its expression widely varied on different tissues and developmental stage, *TCTP* was reported to be regulated in various stresses (Ermolayev et al., 2003; Jones et al., 2006; Vincent et al., 2007; Cao et al., 2010; Li et al., 2010b, 2013; Kim et al., 2012; Wang et al., 2012) and growth regulators (Berkowitz et al., 2008; Liang et al., 2009; Cao et al., 2010; Kim et al., 2012; Li et al., 2013). In accordance with the aforementioned results, *HbTCTP* was regulated by ET, wounding, MeJA, low temperature, high salt, H_2_O_2_ and drought treatments. The *cis*-acting regulatory elements that respond to hormones and stresses were identified in *HbTCTP* promoter, which might explain its expression profiles under hormone and stresses treatments. Although *HbTCTP1* was also regulated by all the aforementioned treatments, the expression patterns of *HbTCTP* evidently differ from that of *HbTCTP1*. Moreover, the *HbTCTP1* transcripts were obviously higher in healthy rubber tree than TPD tree (Li et al., 2013). On the contrary, *HbTCTP* was significantly downregulated in healthy rubber tree. According to the expression patterns of two *TCTP* genes, it is reasonable that there is a functional specialization between *HbTCTP* and *HbTCTP1*.

Hoepflinger et al. (2013) reported that AtTCTP-GFP indicated cytosolic distribution. Mammalian TCTP was predominantly found in cytosol and nucleus *in vivo*, although it was described as a protein functioning in mitochondria (Zhang et al., 2002). In our study, HbTCTP was found to be localized in membrane, cytosol, and nucleus in rice protoplasts, which is consistent with the result that no signal peptide was predicted within HbTCTP protein by using SignalP 4.0 (http://www.cbs.dtu.dk/services/SignalP/). Of course, the subcellular location of HbTCTP need be further validated in rubber tree. TCTP is a multifunctional protein, which is inferred from the fact that it is capable of interacting with diverse targets, including cytoskeletal components, factors involved in cell repair, apoptosis (pro- and antiapoptotic), protein synthesis, and even general metabolism (Amson et al., 2013). In this study, we identified that HbTCTP interacted with HbREF, 17.5 kDa heat shock protein, annexin, and stress-related protein-like. Consisting with our result, Zeng et al. (2011) also reported that HbREF interacted with HbTCTP. REF is necessary for prenyltransferases from a number of sources to add multiple *cis*-isoprene units to rubber molecules (Dennis and Light, 1989). In addition, *HbTCTP* was preferential expression in latex. Therefore, HbTCTP might be involved in rubber biosynthesis (RB) by interacting with HbREF.

An uncompensated oxidative stress occurred on the onset of TPD (Yusof et al., 1995). In TPD tree, the NAD(P)H oxidase activities increased, whereas variable peroxidase and SOD levels decreased (Xu and Xiao, 1988). In addition, Peng et al. (2011) reported that TPD rubber tree indicated typical characteristics of programmed cell death (PCD). Li et al. (2010a) proposed that reactive oxygen species (ROS) metabolism, ubiquitin proteasome pathway (UPP), PCD, and RB pathways might play important roles in TPD. Gébelin et al. (2013) found that the target genes of TPD-related microRNAs were associated with RB, ROS-scavenging systems, and PCD. By identifying and analyzing TPD-related genes in rubber tree bark, Li et al. (2016) further reported that the TPD-related genes significantly enriched in eight GO terms and five KEGG pathways were closely associated with ROS metabolism, PCD, and RB. In this study, the *HbTCTP* transcripts were higher in TPD rubber tree than healthy rubber one. HbTCTP indicated antioxidant activity *in vitro*. Interestingly, HbTCTP also interacted with 17.5 kDa heat shock family protein, and annexin. sHSPs are molecular chaperones that prevent misfolding and irreversible aggregation of their client proteins; moreover sHSPs have roles in differentiation, proteasomal degradation, anti-apoptotic property and development (Bakthisaran et al., 2015). Plant annexins are involved in abiotic stress responses (Divya et al., 2010; Huh et al., 2010) and oxidative stress responses (Richards et al., 2014). Given the aforementioned results, HbTCTP might participate TPD by regulating ROS metabolism, PCD, and RB.

## Conclusion

In summary, *HbTCTP* was regulated by drought, low temperature, high salt, ET, wounding, H_2_O_2_, and MeJA. HbTCTP was localized in the membrane, cytoplasm, and nucleus of rice protoplasts. Being similar to HbTCTP1, HbTCTP indicated antioxidant activity *in vitro*. Interacting with HbREF, 17.5 kDa heat shock protein, annexin, and REF-like stress related protein 1, HbTCTP might be involved in TPD by regulating ROS metabolism, PCD, and RB pathways in rubber tree.

## Author contributions

DL and ZX conceived and designed the experiments. DL wrote the manuscript. ZZ, ZD, and LJ revised the manuscript. ZD and JC performed most of the experiments and data analysis. LJ and PM performed miRNA degradome analysis. ZZ carried out subcellular location analysis. CL, HL, and HY performed experimental samples collection, DNA and RNA extraction. All authors have read and approved the manuscript.

### Conflict of interest statement

The reviewer LZ declared a shared affiliation, though no other collaboration, with one of the authors ZZ to the handling Editor, who ensured that the process nevertheless met the standards of a fair and objective review. The other authors declare that the research was conducted in the absence of any commercial or financial relationships that could be construed as a potential conflict of interest.
